# Polymorphisms in migraine-associated gene, *atp1a2,* and ischemic stroke risk in a biracial population: the genetics of early onset stroke study

**DOI:** 10.1186/2193-1801-2-46

**Published:** 2013-02-11

**Authors:** Andrea M Harriott, Nicole Dueker, Yu-Ching Cheng, Kathleen A Ryan, Jeffrey R O’Connell, O Colin Stine, Patrick F McArdle, Marcella A Wozniak, Barney J Stern, Braxton D Mitchell, Steven J Kittner, John W Cole

**Affiliations:** School of Medicine, University of Maryland, Baltimore, 655 W. Baltimore St, Baltimore, MD 21201 USA; Miller School of Medicine, University of Miami, Miami, FL USA; Veterans Administration Medical Center, Baltimore, MD USA

**Keywords:** Headache, Migraine, Stroke, Genetics, *ATP1A2*, Young

## Abstract

In a recent meta-analysis migraine was associated with a two-fold increase in stroke risk. While the mechanism driving this association is unknown, one intriguing hypothesis is that migraineurs are genetically predisposed to developing ischemic stroke. Mutations in the *ATP1A2* gene are implicated in familial hemiplegic migraine type II and increase the severity of ischemic brain injury in animal models. To further explore these observations, we assessed the association between *ATP1A2* polymorphisms, migraine, and the risk of ischemic stroke in participants of the Genetics of Early-Onset Stroke Study, a population-based case–control study of ischemic stroke among men and women aged 15–49. Using responses to a headache symptoms questionnaire, subjects were classified as having no migraine, or migraine with or without visual aura. Evaluating a total of 134 *ATP1A2* polymorphisms genotyped using a combination of Illumina platforms (Cardiovascular Gene-centric 50 K SNP Array and HumanOmni1-Quad_v1-0_B Bead Chip), only one polymorphism (*rs2070704*) demonstrated a nominally significant association with stroke in an age-, gender-, ethnicity-adjusted model (OR = 0.83, 95% CI = 0.71-0.98, p = 0.025) and in a vascular risk factor model adjusting for age, gender, ethnicity, hypertension, diabetes, smoking, and myocardial infarction (OR = 0.74, 95% CI = 0.63-0.89, p = 0.001). Ethnicity-stratified analyses demonstrated a significant association for *rs2070704* among African-Americans (OR = 0.68, 95% CI = 0.53-0.90, p = 0.005) but not Caucasians (OR = 0.82, 95% CI = 0.64-1.04, p = 0.107). These associations were unchanged when migraine subtypes were included as co-variates. We did not observe an association between *ATP1A2* polymorphisms and migraine. While our results do not demonstrate a strong relationship between *ATP1A2* polymorphisms and migraine associated stroke risk, the results are hypothesis generating and indicate that an association between *ATP1A2* polymorphisms and stroke risk may exist. Additional studies are required.

**Electronic supplementary material:**

The online version of this article (doi:10.1186/2193-1801-2-46) contains supplementary material, which is available to authorized users.

## Background

Although the evidence supporting an etiologic relationship between migraine and ischemic stroke risk is substantial (Cole & Kittner 2010; Sacco et al. 2012; Sacco et al. 2008; Sacco et al. 2006), the precise mechanism(s) driving this relationship remains uncertain. Stroke, which typically affects older individuals, can be targeted for prevention by optimizing well-established standard vascular risk factors such as hypertension, diabetes, and hypercholesterolemia, among others. However, there exist young stroke populations (e.g. < 50 years of age) who have a paucity of established stroke risk factors. One explanation for stroke in this young population is the existence of non-standard risk factors, including migraine headache (Cole & Kittner 2010).

Migraine is a multi-factorial neurological condition characterized by debilitating, recurrent headaches which often affect women and men in younger age groups. Migraine pathogenesis is believed to involve both genetic and environmental factors which ultimately lead to activation of the trigeminovascular system, inflammation, and changes in cerebral blood flow (D’Andrea & Leon 2010). Notably, there is growing evidence that migraine is an independent risk factor for ischemic stroke (Etminan et al. 2005; Lampl & Marecek 2006; MacClellan et al. 2007; Moskowitz & Kurth 2007; Pezzini 2010; Spector et al. 2010; Tzourio et al. 1995; Carolei et al. 1996). In recent meta-analyses; migraine, particularly those associated with aura, significantly increased the risk of stroke by two-fold (Etminan et al. 2005; Spector et al. 2010). Furthermore, this association was most profound in women, persons aged 45 and younger, and those taking oral contraceptives (Etminan et al. 2005). While the mechanism(s) driving this association is (are) unknown, one potential hypothesis is that migraineurs are more susceptible to developing ischemic stroke due to a shared genetic predisposition to both conditions. Consistent with this hypothesis, studies evaluating genetic polymorphisms in the vasoconstrictor endothelin-1 gene (*EDN1*) were associated with increased risk of stroke risk as well as migraine (Etminan et al. 2005; MacClellan et al. 2007; MacClellan et al. 2009; Tikka-Kleemola et al. 2009). While a number of additional candidate genes have been postulated to increase stroke risk via such shared mechanisms, at this time the definitive genetic links between migraine and stroke remain elusive.

Familial hemiplegic migraine (FHM) is a rare inherited form of migraine that presents with asymmetric neurological deficits (Tavraz et al. 2008). Several gene mutations have been implicated in the pathogenesis of FHM including genetic mutations in *ATP1A2*, *CACNA1A* and *SCN1A*. These genes encode for a neuronal and glial Na^+^/K^+^ ATPase; a voltage gated Ca^2+^, and; a Na^+^ channel respectively. Interestingly, *ATP1A2* gene mutations result in degeneration of the amygdala and pyriform cortex (Ikeda et al. 2003). Furthermore, failure of the Na^+^/K^+^ ATPase is involved in ischemic brain injury and glutamatergic excitotoxicity (Stys 2004; Wang & Qin 2010). Such observations suggest that the *ATP1A2* gene may influence stroke risk and that some migraineurs may be particularly predisposed. To test this hypothesis we assessed the association between *ATP1A2* single nucleotide polymorphisms (SNPs), migraine, and the risk of ischemic stroke in a previously collected biracial case–control sample of young-onset ischemic stroke.

## Results

### Demographic and clinical characteristics of the population

Characteristics of the 1737 GEOS study participants (830 cases - 43% African-American and 907 controls - 39% African-American) are summarized in Table 1. The population included men (56%) and women (44%) aged 15–49 with a median age of 43 in the cases and 41 in the controls. Cases were more likely than controls to report having prevalent hypertension, diabetes, and myocardial infarction and to being current smokers. Among women with stroke, cases reported a greater prevalence of oral contraceptive use. Additionally, cases reported more Migraine with Aura (27%) as compared to controls (21%).

**Table 1:** **Demographic and clinical characteristics of cases/controls**

	Cases (n = 830)	Controls (n = 907)	P
Age*	43 (39–47)	41 (36–44)	<0.001
Female	354 (43%)	407 (45%)	0.421
Black	353 (43%)	354 (39%)	0.207
HTN	371 (45%)	181 (20%)	<0.001
MI	62 (7.5%)	14 (1.5%)	<0.001
DM	147 (18%)	55 (6%)	<0.001
Smoking	348 (42%)	257 (28%)	<0.001
OC	57 (16%)	34 (8%)	<0.001
Migraine			
MWA	226 (27%)	193 (21%)	0.005
MWoA	56 (7%)	73 (8%)	0.346

*HTN*, Hypertension *MI*, Myocardial Infarction *DM*, Diabetes Mellitus Type II *OC*, Oral Contraceptive use. * Age is expressed as median (25th-75th percentile).

### *ATP1A2* SNPs association studies with stroke

**Figure 1:**
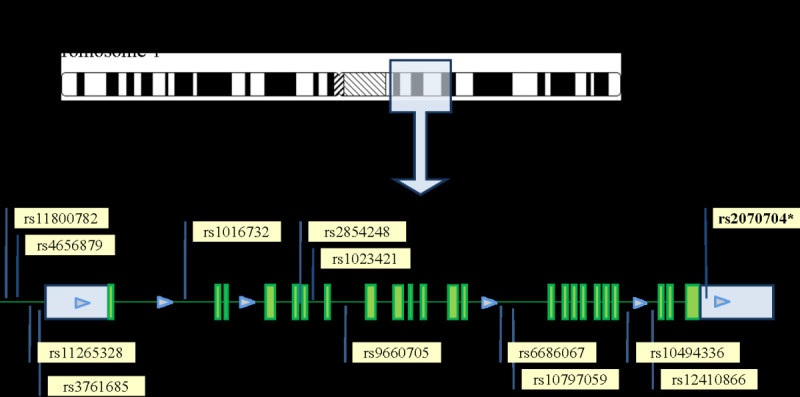
**A. The*ATP1A2*gene is located on chromosome 1 and contains 23 exons. B**. Denotes the location and rs numbers of the thirteen *ATP1A2* SNPs genotyped as per the fixed content of the Illumina Cardiovascular Gene-centric 50 K SNP Array (ITMAT-Broad-CARe array) (Keating et al. 2008).

Association analyses between thirteen *ATP1A2* SNPs (see Figure 1) on ITMAT-Broad-CARe array and stroke were initially performed. The minor allele frequencies compared between cases and controls as stratified by ethnicity are listed in Table 2.

**Table 2:** ***ATP1A2*** **allele frequency of cases and controls stratified by race**

		Minor allele frequency			
		Cases		Controls	
***ATP1A2*** SNP*	Position	Black	White	Black	White
rs11800782_**A**/G	160081369	0.014	0.037	0.004	0.053
rs4656879_**C**/T	160083012	0.298	0.140	0.322	0.149
rs11265328_**A**/G	160083544	0.025	0.099	0.025	0.106
rs3761685_**C**/T	160084643	0.024	0.128	0.027	0.125
rs1016732_**A**/G	160086968	0.113	0.173	0.093	0.179
rs2854248_**A**/G	160093927	0.459	0.359	0.477	0.359
rs1023421_**A**/G	160094644	0.492	0.382	0.448	0.377
rs9660705_C/**T**	160096810	0.065	0.000	0.063	0.000
rs6686067 _**C**/T	160102060	0.075	0.138	0.065	0.153
rs10797059_**A**/G	160102256	0.234	0.138	0.264	0.153
rs10494336_**A**/G	160107588	0.169	0.098	0.164	0.114
rs12410866 _C/**T**	160108803	0.025	0.115	0.023	0.130
rs2070704_A/**G**	160112138	0.228	0.195	0.287	0.205

* minor allele bolded.

Of the thirteen SNPs examined, only *rs2070704* was associated with stroke. As demonstrated in Table 3, the G allele confers protection against stroke in the minimally adjusted model (OR 0.83, 95% CI 0.7-0.9, p = 0.025) with a stronger protective effect in the risk factor fully adjusted model (OR 0.74, 95% CI 0.6-0.9, p < 0.001). Stratified analyses by ethnicity (including age, gender, hypertension, diabetes, myocardial infarction, smoking, and oral contraceptive use as covariates) demonstrated an association in the African-American population (OR 0.68, 95% CI 0.5-0.9, p = 0.005), but not the Caucasian population (OR 0.82, 95% CI 0.6-1.0, p = 0.107). Gender stratified analyses demonstrated the association to be strongest among African-American males (OR 0.55, 95% CI 0.4-0.8, p = 0.002). Correcting for multiple comparisons including the 13 SNPs and assuming a p-value = 0.004 (0.05/13) as significant; our results for the entire population (p < 0.001) remain nominally significant, however our results for the African-American population (p = 0.005) do not.

**Table 3:** **Effect of SNP** ***rs2070704*** **on Ischemic Stroke Risk**

			Minimally adjusted model†		Full model‡	
Subjects	Cases/controls	MAF (G allele) cases/controls	OR (95%CI)	P	OR (95%CI)	P
Entire Population	829/899*	0.203/0.238	0.83 (0.7-0.9)	0.025	0.74(0.6-0.9)	0.001
***Stratified***						
Blacks	355/351	0.225/0.288	0.71 (0.5-0.9)	0.007	0.68 (0.5-0.9)	0.005
Black Female	157/160	0.229/0.259	0.88 (0.6-1.3)	0.490	0.83 (0.6-1.2)	0.311
Black Male	198/191	0.215/0.312	0.58 (0.4-0.8)	0.002	0.55 (0.4-0.8)	0.002
Whites	431/500	0.188/0.205	0.93 (0.7-1.2)	0.499	0.82 (0.6-1.0)	0.107
White Female	165/215	0.210/0.209	1.03 (0.7-1.5)	0.867	0.88 (0.6-1.3)	0.530
White Male	266/285	0.174/0.202	0.85 (0.6-1.2)	0.291	0.80 (0.6-1.0)	0.093

†: Age, Gender, Race.

‡: Age, Gender, HTN, DM, MI, Smoking, Oral Contraceptive use, Race.

* Of the 830 cases and 907 controls, 3 cases and 6 controls failed genotyping at rs2070704.

Implementing the same regression models we then evaluated the *ATP1A2* GWAS data on these same study subjects. As described in the Methods section, this larger data set included 130 SNPs in the *ATP1A2* gene, 9 of which overlapped with the ITMAT-Broad-CARe array data. As consistent with our ITMAT-Broad-CARe array data, *rs2070704* demonstrated an association. This was the only SNP of the 130 SNPs evaluated that demonstrated an association. Ethnicity stratified analyses evaluating ischemic stroke risk associated with *rs2070704* likewise demonstrated a significant association among African-Americans (N = 733, MAF = 0.29, OR = 0.66, p = 0.0009) but not among European-Americans (N = 946, MAF = 0.20, OR = 0.91, p = 0.40). Correcting for multiple comparisons including all 134 SNPs evaluated, and assuming a p-value = 0.0004 (0.05/134) as significant, none of our results remain significant.

Analyses of *rs2070704* by stroke subtype in the combined population only demonstrated an association among strokes of undetermined etiology (OR 0.76, p = 0.009). Ethnicity stratified analyses demonstrated no subtype specific associations among Caucasians. Among African-Americans the protective effect of *rs2070704* was greatest in the large-artery atherosclerotic subtype (OR = 0.18, p = 0.002) as compared to cardio-embolic (OR = 0.61, p = 0.029), small vessel (OR = 0.71, p = 0.130) and strokes of undetermined etiology (OR = 0.71, p = 0.030).

### Migraine-stratified Analyses between *rs2070704* SNP and stroke

Utilizing the fully adjusted model we added migraine status as a co-variate. The prediction for this analysis was that if migraine mediates the association between the *rs2070704* SNP and stroke, adding migraine as a co-variate should eliminate this association. However, when migraine was included in the model, the association between *rs2070704* and stroke (OR 0.75, 95% CI 0.6-0.89, p = 0.0009; G-allele case/control: 0.221/0.260) remained essentially unchanged. We then performed a restricted analysis adding either migraine without aura or migraine with aura as a co-variate. Again, there was no change in the association; migraine without aura (OR 0.71, 95% CI 0.6-0.88, p = 0.0012) and migraine with aura (OR 0.76, 95% CI 0.6-0.9, p = 0.0024), indicating that migraine status does not mediate the relationship between this SNP and stroke risk.

Lastly, the association between *ATP1A2* SNPs and migraine status was tested using a fully adjusted logistic regression model accounting for the same risk factors. We did not observe an association between any *ATP1A2* polymorphisms and migraine as defined by this study.

## Discussion

Our analyses of the *ATP1A2* gene demonstrated one polymorphism that was nominally associated with ischemic stroke; however this association did not persist with correction for multiple testing, nor was this association mediated by migraine status. Our study was motivated by the fact that prior studies have shown that the *ATP1A2* gene is implicated in rare forms of migraine (familial hemiplegic migraine type II) which has features typical of migraine with aura in addition to features consistent with transient brain ischemia (Hansen et al. 2011; Mourand et al. 2012; Eikermann-Haerter et al. 2012).

**Figure 2:**
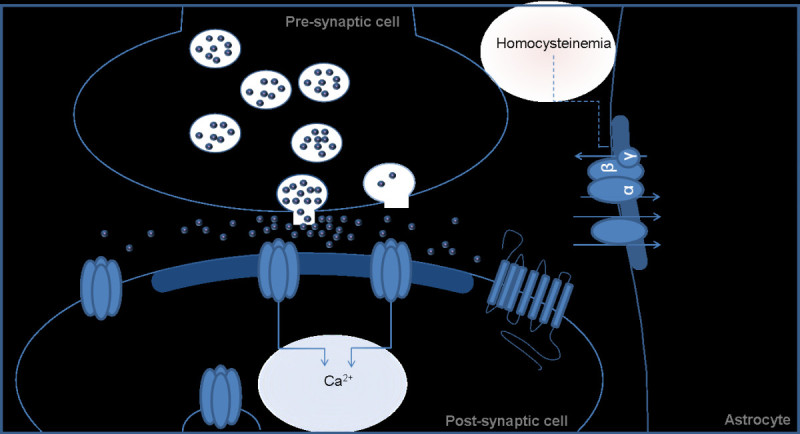
**Image demonstrating potential mechanisms relating how altered Na**^**+**^**/K**^**+**^**ATPase (as coded by*ATP1A2)*function could be related to stroke risk.** Decreased Na^+^/K^+^ ATPase activity leads to: altered ionic (Na^+^, K^+^) gradients resulting in excess intracellular Ca^2+^ (Danbolt 2001); impaired K^+^ channel-mediated membrane repolarization (Luo et al. 2004; Matsuda et al. 2001), and; elevated glutamate concentrations at the synaptic cleft (Rose et al. 2009). Elevated homocysteine has been shown to decrease Na^+^/K^+^ ATPase function (Machado et al. 2011; Streck et al. 2002).

*ATP1A2* encodes for the α_2_ subunit of Na^+^/K^+^ ATPases (Pietrobon 2007). Figure 2 highlights several potential mechanisms by which dysfunction of Na^+^/K^+^ ATPases could modify stroke risk. Glial Na^+^/K^+^ ATPases are responsible for generating and maintaining ionic electrochemical gradients across plasma membranes (Danbolt 2001). Data from experimental animal models suggest that Na^+^/K^+^ ATPase dysfunction can dissipate both Na^+^ and K^+^ gradients increasing the potential for neuronal excitoxicity. Dissipation of the Na^+^ gradient limits the effectiveness of Na^+^/Ca^2+^ transporters resulting in excess accumulation of intracellular Ca^2+^. Similarly, dissipation of the K^+^ gradient impairs K^+^ channel-mediated membrane repolarization (Luo et al. 2004; Matsuda et al. 2001). At excitatory glutamatergic synapses, glutamate re-uptake is coupled to Na^+^ transport. Therefore, decreases in Na^+^/K^+^ ATPase function raise glutamate concentration at the synaptic cleft inducing glutamate-mediated excitotoxicity and cell death (Rose et al. 2009). Interestingly, in animal models of ischemic stroke, decreases in Na^+^/K^+^ ATPase function and expression appear to play a significant role in neuro-degeneration during cerebral infarction (Martin et al. 1994; Park & Jung 2010; Sheldon & Robinson 2007). Prior genetic studies demonstrate an association between the *MTHFR* C677T polymorphism and stroke that appear to be related to increased homocysteine concentration (Casas et al. 2005), which in animal models is linked to decreased Na^+^/K^+^ ATPase function (Machado et al. 2011; Streck et al. 2002). As such, these data provide evidence that there may be a common pathway between the protein product of previously identified stroke-related genes and the *ATP1A2* gene. These data also allude to a potential combinatorial effect of multiple genes which could play an intricate inter-related role in stroke pathogenesis (Figure 2).

The presence of migraine has been shown to significantly increase the risk of ischemic stroke (Cole & Kittner 2010; Etminan et al. 2005; Lampl & Marecek 2006; MacClellan et al. 2007; Hansen et al. 2011; Eikermann-Haerter et al. 2012). This association is thought to be in part related to a common genetic susceptibility; however, there are few studies linking migraine-related genes with stroke risk. One example is endothelin, a potent vasoconstrictor, which has been shown to be increased in migraineurs in both the ictal and interictal periods (Lo et al. 2005). A recent case–control study demonstrated that polymorphisms in the *Endothelin 1* gene increase stroke risk (MacClellan et al. 2009), and in animal models of middle cerebral artery occlusion, over-expression of endothelin produced increased brain edema and ischemic brain injury (Lo et al. 2005).

We used the strategy of studying the early-onset form of ischemic stroke to identify genetic variants associated with this complex disease for two reasons. First, the early-onset form is thought to have a reduced cumulative burden of standard risk factors, although they were highly prevalent in our early-onset population as demonstrated in Table 1. Second, genetic components are thought to have a greater contribution to disease risk in this population (MacClellan et al. 2006). While it is possible that genetic variants associated with early-onset stroke may have little relevance to late-onset stroke, it is also possible that studying the early-onset form of diseases may reveal important insights about stroke pathogenesis, potentially implicating genes and pathways relevant to late-onset disease even though the etiologic variants may differ.

There are several limitations to our study. The absence of robust associations may be due to the limited sample sizes (as stratified by gender, ethnicity, migraine status, stroke subtype) which provided insufficient power to detect causal variants with small effect sizes (e.g. less than 80% power to detect genetic risk effect less than 1.23). Alternatively, one can argue that the early-onset form of ischemic stroke may be more likely to be caused by rare variants with larger effects (and higher penetrance). Such rare variants are generally not well covered on currently available genotyping platforms and, thus, would be difficult to detect using the common variants as provided by GWAS data. Additionally, in our study population, a greater percentage of subjects had migraine with aura compared to migraine without aura which differs from prior epidemiological studies (D’Andrea et al. 2011). Of note, our headache questionnaire was not validated for migraine, and the migraine criteria implemented for this study were slightly different than those of the International Headache Society (IHS). Both criteria use 5 attacks for migraine without aura and 2 attacks for migraine with aura; accompanied by nausea, vomiting, sensitivity to light and/or sound. The migraine with aura group also reported the presence of photopsia, fortification spectra or scotoma with the headache. However, IHS criteria include a temporal relationship between headache onset and aura, non-visual aura and duration of headache. Despite these differences, but similar to prior studies (MacClellan et al. 2007), there was a greater percentage of migraineurs with aura in cases as compared with controls. Lastly, our study may be subject to recall bias, particularly among the case subjects, who may have responded to the questionnaire including headache symptoms, as far out as 3 years following their stroke.

## Conclusions

Our data indicate that common variants in the *ATP1A2* gene do not play a large role in early-onset ischemic stroke risk or migraine headache. While we did identify one *ATP1A2* polymorphism, *rs2070704,* that may play a role in early-onset stroke pathogenesis, particularly among African-Americans, migraine did not mediate this affect. Additional studies will be required to confirm our findings in early-onset stroke and to explore the relationship between *ATP1A2* and older-onset stroke.

## Methods

### Study population and design

Data from the retrospective case–control Genetics of Early-Onset Stroke (GEOS) Study was used to determine if SNPs in the ATP1A2 gene confer susceptibility to stroke and if this susceptibility is mediated by migraine. Subjects included 830 cases of first ever ischemic stroke and 907 age, ethnicity and gender-matched controls that were free of ischemic stroke. Study recruitment and data collection occurred in three waves with the initial recruitment for females occurring between 1992 and 1996, and again between 2001 and 2003. Male subjects were recruited between 2003 and 2007. In all three waves, cases were hospitalized with a first cerebral infarction identified by discharge surveillance from one of 59 hospitals in the greater Baltimore-Washington area and direct referral from regional neurologists. The methods for discharge surveillance, chart abstraction, case adjudication, and assignment of probable and possible underlying causes have been previously described elsewhere (MacClellan et al. 2007; MacClellan et al. 2009; MacClellan et al. 2006). Control subjects were subjects with no history of stroke identified by random-digit dialing and were matched to cases by age (within ten years) and geographic region of residence. The first wave of female recruitment included cases ages 15–44 years recruited within one year of stroke and was designed with a 1:2 case to control ratio. The second wave of female recruitment and the sole wave of male recruitment included cases ages 15–49 recruited within three years of stroke and designed with a 1:1 case to control ratio. For all study periods, additional cases were recruited after completion of control recruitment.

All study subjects signed Institutional Review Board approved written informed consent forms. Subjects were excluded from the study if they had genetic or other known causes for their stroke that would impair detection of new genetic associations. These conditions included: sickle cell disease, thalassemia disease, central nervous system vasculitis by angiogram and clinical criteria, endocarditis, neurosyphillis, mechanical heart valve, post-radiation arteriopathy, and cocaine use within 48 hours of stroke. Data on historical risk factors among both cases and controls was collected by standardized interview. Using responses to a headache symptoms questionnaire, all subjects were classified as having no migraine, or migraine with or without visual aura. Subjects were classified as having migraine *with* visual aura if they: (1) reported ever seeing spots, lines, or flashing lights around the time of their probable migraine; or (2) if they reported ever experiencing loss of vision and also reported a frequency of probable migraine with visual aura of at least twice per year. Subjects were identified as having probable migraine *without* visual aura if they reported no history of visual aura and reported nausea, vomiting, or sensitivity to light during a probable migraine and probable migraine frequency of at least 5 times per year. Traditional stroke risk factors and other study variables, including age, ethnicity, history of hypertension, diabetes, myocardial infarction (MI), and current smoking status (defined as use within one month prior to event for cases and a comparable reference time for controls), were also collected during a standardized interview. The abstracted hospital records of cases were reviewed and adjudicated for ischemic stroke subtype by a pair of vascular neurologists as previously described (MacClellan et al. 2007) with disagreements resolved by a third neurologist. The ischemic stroke subtype classification system retains information on all probable and possible causes, and is reducible to the more widely used TOAST system (Adams et al. 1993) that assigns each case to a single category. As previously described, genomic DNA was isolated from a variety of sample types, including cell line, whole blood, mouth wash, and buccal swab among all case and controls subjects (Hamedani et al. 2011).

### Single nucleotide polymorphism selection

As demonstrated in Figure 1, the *ATP1A2* gene is located on chromosome 1q21-23, is 27.8 kb in size, and contains 23 exons (Genomic coordinates (GRCh37): 1:160,085,519 - 160,113,380). Our initial analyses included the 13 *ATP1A2* SNPs distributed throughout the gene (denoted in Figure 1) that were available from the fixed content on the Illumina Cardiovascular Gene-centric 50 K SNP Array (ITMAT-Broad-CARe array).(Keating et al. 2008) This fixed content array includes 49,094 SNPs from ~2,000 loci and had been previously implemented on our 830 case and 907 control subjects. Genotyping was performed at the Institute for Translational Medicine and Therapeutics (ITMAT), University of Pennsylvania.

Shortly after completing our analyses, additional *ATP1A2* genotype data became available on these same study subjects as provided by a genome-wide scan (Cheng et al. 2011) implementing the Illumina HumanOmni1-Quad_v1-0_B Bead Chip. These data provided more extensive coverage of *ATP1A2,* including 130 SNPs, of which 9 SNPs overlapped between the two fixed-content Illumina arrays. Comparing genotype calls between the two arrays demonstrated >99% concordance among the 9 overlapping SNPs. Inclusive of both platforms, a total of 134 different SNPs were evaluated; SNPs were required to have a minor allele frequency greater than 5% in at least one of the two ethnicities (i.e. Caucasians and/or African-Americans).

### Statistical analysis

Demographic and risk factor data between cases and controls were compared with either a *t*-test or *Χ*^2^ test using Sigma Plot v11.0 software. For primary analyses, the entire population and then an ethnicity-stratified, minimally adjusted additive logistic regression model was used taking into account age and gender to test the association between each SNP and stroke (CC = SNP + age + gender where CC is case/control status). Each SNP was then also evaluated utilizing a fully adjusted model that included vascular risk factors; hypertension, prior myocardial infarction, diabetes, oral contraceptive use, and smoking. Analyses stratified by stroke subtype (small vessel - lacunar; large vessel - atherosclerotic; cardioembolic; stroke of unknown etiology - cryptogenic) were performed including age, ethnicity, and gender as covariates. SNPs demonstrating an association with stroke were then further analyzed incorporating a variable for the presence or absence of migraine, as well as the presence or absence of migraine with aura. A fully adjusted model was also used to test the association between *ATP1A2* polymorphisms and migraine irrespective of the presence or absence of stroke. All logistic regression analyses were performed using SAS v9.2 software. Odds Ratios (OR) are listed for minor alleles and a p < 0.05 was considered nominally significant. Multiple comparisons were assessed using a Bonferroni correction among the initial 13 SNPs and then among the larger panel of 134 SNPs. Power calculations (using CaTS Power Calculator @ http://www.sph.umich.edu/csg/abecasis/cats/) indicated that our sample size of 830 cases and 907 controls provided 80% power to detect ORs ranging 1.23–1.49 for allele frequencies ranging 5–50% at an α = 0.05.

## Authors’ original submitted files for images

Below are the links to the authors’ original submitted files for images. Authors’ original file for figure 1 Authors’ original file for figure 2
