# Supplementary figures and images for: Polymorphisms in migraine-associated gene, atp1a2, and ischemic stroke risk in a biracial population: the genetics of early onset stroke study
Source: Springerplus. 2013 Feb 11;2(1):46. doi: 10.1186/2193-1801-2-46 (PMC3582818; doi:10.1186/2193-1801-2-46)

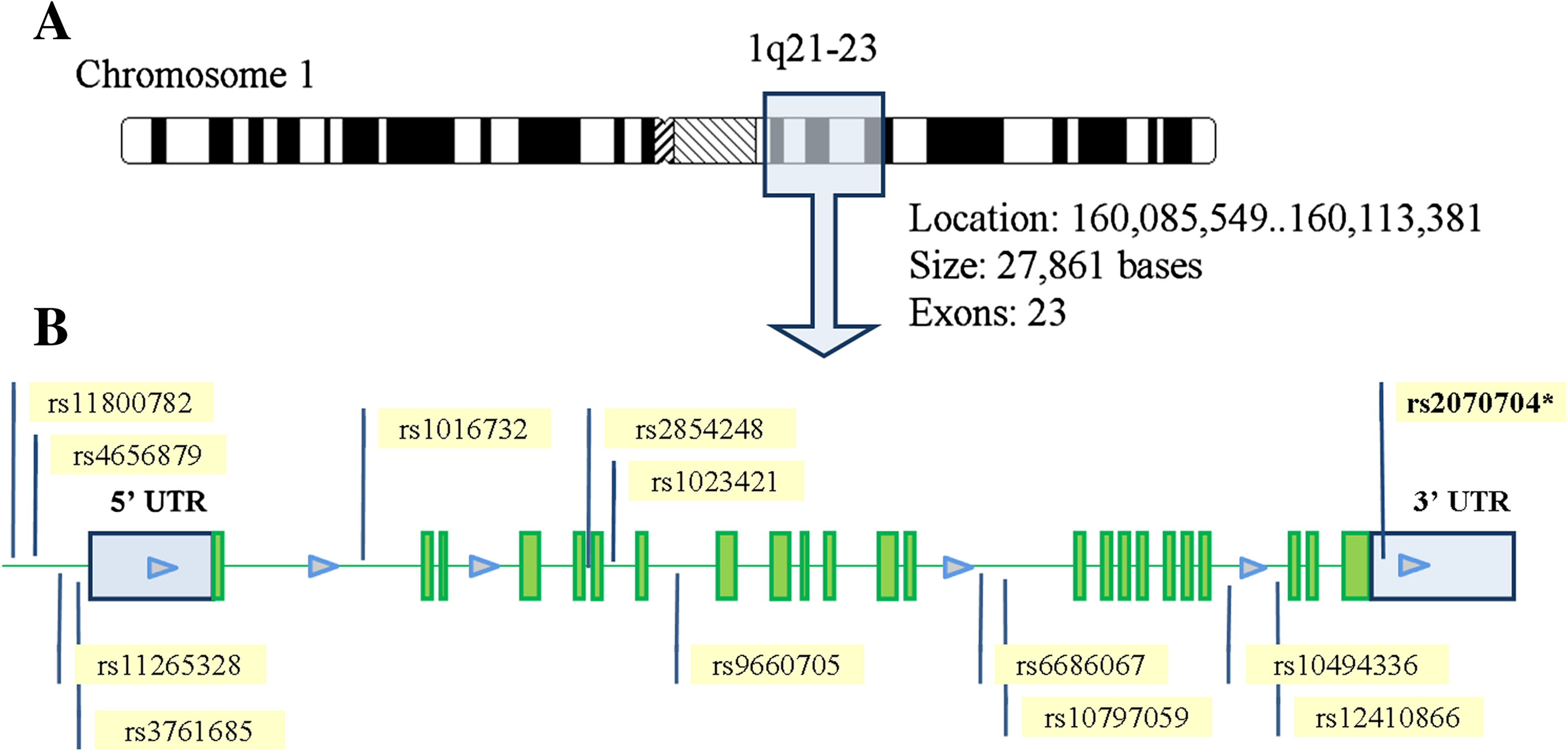

Supplement: Supplementary file 1 — Authors’ original file for figure 1 [file 40064_2013_95_MOESM1_ESM.tiff]

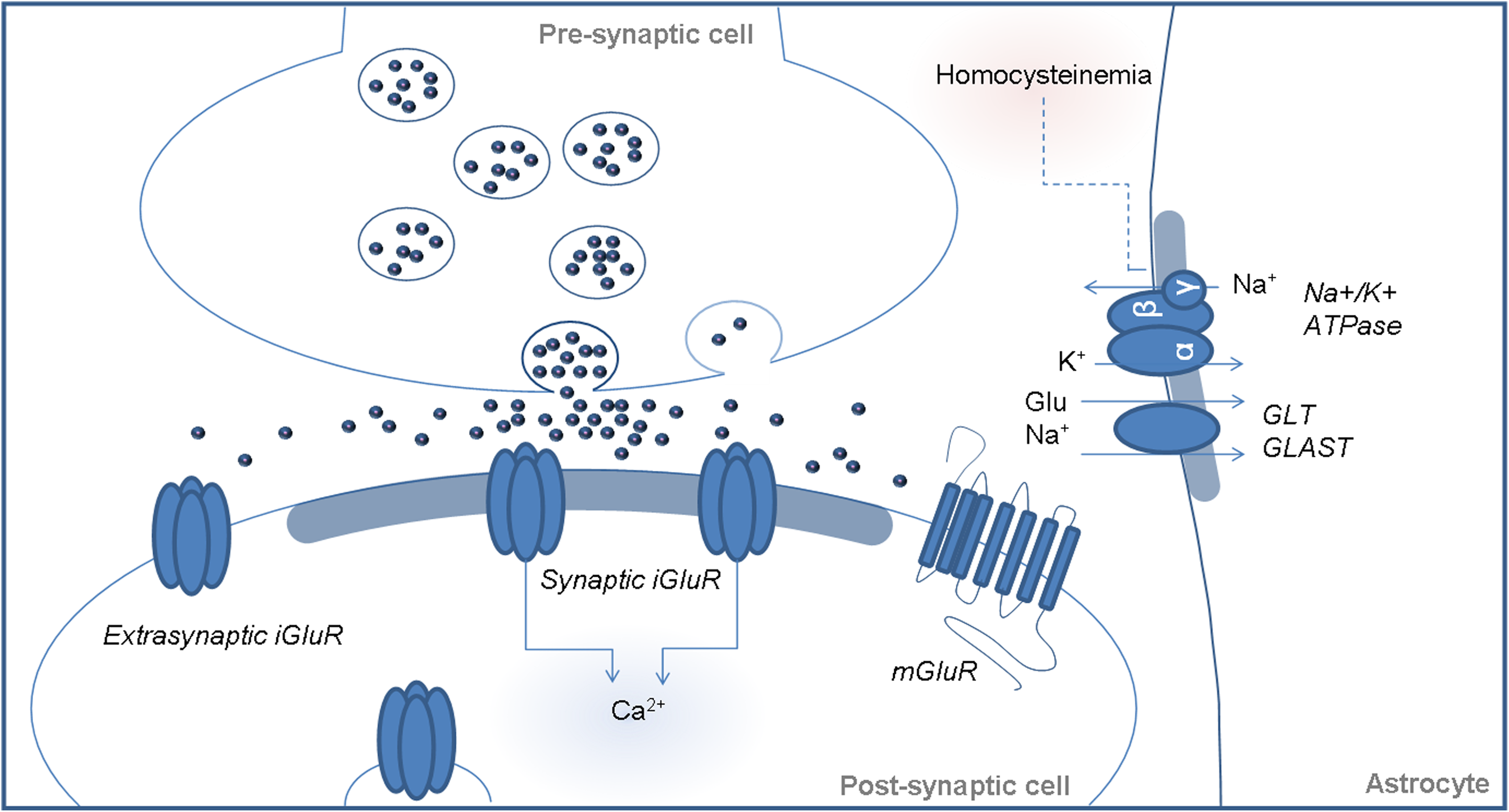

Supplement: Supplementary file 2 — Authors’ original file for figure 2 [file 40064_2013_95_MOESM2_ESM.tiff]
